# A systematic review of consumers’ knowledge, attitudes and experiences of primary health professionals’ role in genomic medicine

**DOI:** 10.1038/s41431-025-01904-y

**Published:** 2025-07-02

**Authors:** Samran Sheriff, Maryam Vizheh, Romika Patel, Samantha Spanos, Klay Lamprell, Jeffrey Braithwaite, Janet C. Long

**Affiliations:** Centre for Healthcare Resilience and Implementation Science, Australian Institute of Health Innovation, Macquarie University, Sydney, NSW Australia

**Keywords:** Genetic testing, Genetic services

## Abstract

The integration of genetic testing into primary care is influencing healthcare practices, yet little is known about consumers’ knowledge, attitudes, and experiences with genetic testing services or the practitioners who provide them. This systematic review synthesizes peer-reviewed studies on consumers’ perspectives regarding the role of primary health professionals in delivering genomic medicine in primary care settings. Six databases (PubMed, Scopus, Embase, CINAHL, Cochrane Library and PsycINFO) were systematically searched. Inclusion criteria focused on studies that addressed consumers’ knowledge, attitudes, and experiences related to Primary Care Providers’ (PCP) roles in genomic medicine. Data relevant to the review objective, including key article characteristics, barriers and facilitators of implementation, and recommendations for advancement or optimisation, were extracted and analysed using thematic analysis. We reviewed 19 studies meeting the inclusion criteria involving 3557 participants. Thematic analysis identified two overarching themes: consumer views on genomic testing irrespective of setting, comprising three sub-themes, and consumer views on genomic testing in the primary care setting, comprising four sub-themes. Consumers’ trust in PCPs as familiar and approachable professionals was a major concern. Consumers often reported positive experiences when PCPs were well-informed and communicative, but negative experiences were common when there was a perceived lack of knowledge or confidence from the PCP. As reported in other healthcare settings, concerns about privacy, data security, and the cost of genomic testing were also prominent. Integrating genomic medicine into primary care requires trust-building between PCPs and consumers, enhancing PCP education and resources, addressing privacy and cost concerns and strengthening collaboration with genetic specialists to improve consumer experiences.

## Introduction

The application of genomic and genetic information in clinical practice is rapidly expanding as advances in screening, diagnosis, and treatment of genetic conditions continue to evolve [1]. Whether testing single genes or whole genomes, advances in molecular genetics have made genomic applications more affordable, accurate, and faster, facilitating their adoption across varied clinical settings. Regulatory and legal frameworks [2] are developing in parallel, designed to protect privacy and prevent discrimination, and ensure responsible use of genetic information [3].

Primary care serves as the first point of contact in the healthcare system, offering prevention (e.g., immunisations), diagnosis and treatment of acute illness (e.g., urinary tract infections, minor injuries), and chronic disease management (e.g., asthma, arthritis). The characteristics and services of primary care may vary across health systems, in terms of funding models, physical layout, scope of practice and integration with other services, shaping provider roles and consumer expectations. In some countries, such as the United Kingdom (UK), Canada and Australia, primary care is predominantly funded by universal insurance schemes. In other countries, (e.g., United States [US]) a fee for service or insurance coverage may apply [4, 5]. Family physicians, general practitioners (GPs), or nurse practitioners may work independently or lead a multidisciplinary team, depending on the country and healthcare system.

Genetic testing in the primary care setting covers a range of genetic and genomic applications including testing for suspected conditions (e.g. Haemochromatosis [6], Familial Hypercholesterolaemia [7] and sickle cell anaemia [8]), screening for cancer risk including hereditary cancers, and reproductive genetic carrier screening (RGCS) [9].

More advanced applications, such as polygenic risk scores and comprehensive genetic testing strategies like genome-wide sequencing, largely remain limited to research settings or specialist genetic services. The increasing availability and adoption of direct to consumer (DTC) genetic tests have also had implications for primary care, despite not being ordered by primary care physicians. Clinicians are frequently required to interpret results and address consumer concerns, influencing clinical practice and consumer expectations [10]. These tests are outside the scope of this review.

Mainstreaming of genetic testing (i.e., where non-genetic professionals take on roles such as ordering, testing and counselling) has been facilitated by updated clinical guidelines and the introduction of government-subsidised genetic testing services in some countries (e.g., Medicare rebatable reproductive genetic carrier screens in Australia) [11]. This has allowed primary health practitioners to offer services that were once limited to specialised genetic professionals [12]. There are, however, persistent barriers to the uptake of genetic and genomic testing in primary health settings including lack of awareness amongst healthcare providers and the general community of the availability and indications for testing, the costs involved, and concerns surrounding privacy [3, 4]. Some studies have shown that primary care providers (PCPs) perceive that they have inadequate genetic education and training to manage consumer questions and concerns about the risks, implications and ethical issues related to genetic and genomic testing [13].

Other studies have found that consumers (individuals who directly access and engage with health services, products, or information) report low levels of awareness of genetic risk factors, poor knowledge of the field of genetics and lack of clarity on how to access genetic services in healthcare [14, 15]. Genetic knowledge and deterministic beliefs, which include assumptions that a specific factor, often genetic, solely or largely determines an outcome, disregarding the influence of environmental, lifestyle, or other modifying factors, were the strongest predictors of whether a consumer was willing to undergo genetic testing [16]. Lower levels of education, older age and individuals who belong to an ethnic minority tend to have lower knowledge and understanding of genetics and are likely to develop a pessimistic view around genetic results. Additionally, individuals from disadvantaged socioeconomic backgrounds may face similar challenges due to limited access to genetic education and healthcare resources [9]. Consumer surveys assessing consumer knowledge and attitudes towards genetic testing in any setting indicate that consumers perceive genetic testing as being more important in the future than it is currently [17].

As genomic applications expand in primary healthcare, understanding consumer knowledge, attitudes, and experiences with genetic testing is critical for its successful implementation. While existing research has largely focused on healthcare practitioners particularly genetic professionals there is limited literature on consumer perspectives in this setting [9]. RGCS is a well-studied example, including its implementation in primary care [9]. However, to our knowledge, no systematic review has examined consumer knowledge and attitudes toward genetic applications in primary healthcare. This review aims to address this gap by synthesising peer-reviewed studies on consumers’ perspectives regarding the role of primary health professionals in genomic medicine.

## Methods

This study was conducted and reported in accordance with the Preferred Reporting Items for Systematic Reviews and Meta-Analyses (PRISMA) statement and the Cochrane Handbook guidelines [18, 19].

### Registration and protocol

This systematic review followed a registered protocol on PROSPERO (CRD42024533912).

### Search strategy

Two team members (MV, SSp) developed comprehensive search strategies using a combination of medical subject headings (MeSH) and key words. Subject headings included ‘genetic testing’, ‘primary healthcare’ and ‘general practice’, and key words included attitude*, belief*, experience*, knowledge*, perception*, and perspective* (see Supplementary Fig. 1 for full search strategy). Six databases (PubMed, Scopus, Embase, CINAHL, Cochrane Library and PsycINFO) were systematically searched for articles published since database inception until February 2024. Searches were limited to publications written in English and focusing on human populations. Search terms were used in combination across the various databases, and subject headings adapted to the indexing specifications of each database. The reference lists of relevant reviews were also screened so that additional relevant studies could be included.

Database search results were uploaded to Rayyan software [20], and duplicates were identified and removed. Titles and abstracts were screened for eligibility within Rayyan according to inclusion and exclusion criteria, with 10% of references independently screened by team members (JL, MV, RP, KL, SSp and SS) to ensure inter-rater reliability, using the blinding function in Rayyan. The remaining 90% of articles were then apportioned between authors and independently screened. The full texts of articles deemed potentially relevant were then screened based on inclusion and exclusion criteria (Table 1). One author (SS) hand searched the reference lists of included papers and relevant systemic reviews identified during the screening phase to identify eligible studies in discussion with a second author (SSp). Any conflicts were resolved in discussion by the authors, with JB available as arbiter.

**Table 1:** Inclusion and exclusion criteria for the systematic review.

Inclusion criteria	Exclusion Criteria
English language	Not in English
Peer reviewed empirical studies – (qualitative, quantitative and mixed methods)	Commentaries, letters, editorials, conference abstracts, perspectives, reviews
Studies evaluating consumer attitudes, knowledge, perceptions, or experiences associated with genetic or genomic testing or screening (e.g., RGCS NIPT, PGx)	Not focused on genetics testing or not focused on primary care providers playing a main role in diagnosis, treatment or management Studies looking at views of hypothetical testing
Studies focused on genetic applications used by primary care providers including general practice, midwifery and nurse practitioners	Other settings for genetic and genomic applications.

*NIPT* Non-invasive prenatal test, *PGx* Pharmacogenetics, *RGCS* Reproductive genetic carrier screening.

### Data extraction

The authors (SSh, JL, MV, RP, KL and SSp) extracted basic information from the included studies using a custom designed Microsoft Excel sheet that was trialled and piloted with a subset of the included articles. Information extracted included: first author and year of publication, country of study, study aim, study design and methodology (qualitative, quantitative and mixed methods), health condition, and setting (public, private, family physician or other) type of genetic testing, and participant demographics (e.g., sample size, gender, age, education level and ethnicity).

### Data analysis and coding approach

Two authors (SSh, JL) worked independently to retrieve data from the studies using a piloted data extraction sheet (Microsoft Excel) to extract and code data concurrently. The extraction primarily consisted of qualitative data (e.g. textual descriptions, survey responses), which were verified by the second author (JL) to ensure consistency and accuracy. The authors followed a process of reading the full text of each article, identifying relevant findings and completing extraction. The coding process was managed using Microsoft Excel, with the codes applied in a mixed deductive and inductive manner. First, preliminary codes were developed by the study authors, which were based on previously reported consumers’ attitudes toward genetic testing in a variety of settings [15, 21]. Additionally, new codes were developed inductively based on the content expertise of the research team to capture themes unique to consumer attitudes toward genetic testing in the primary care setting, given the dearth of literature in this particular field. After coding, the authors compared their independently applied codes and synthesised them into cross cutting themes. After synthesising the codes, the findings were discussed with the full research team for validation, ensuring the robustness and reliability of the analysis. This coding approach followed the principles of thematic analysis as outlined by Braun and Clarke [22].

Themes unique to consumer attitudes toward genetic testing in the primary care setting were grouped separately to reflect the particular context of primary care. A data-based convergent synthesis was also employed [23], where quantitative data were transformed into categories or themes and summarised using narrative techniques [24]. For example, in the case of survey studies, the prose summaries provided by the study authors were cross checked and verified with the raw data reported in the study. This data was then analysed as qualitative information. It is important to note that all the survey data used in the theme development were self-reported and subjective in nature, rather than being based on clinical outcomes thus suiting this methodology well.

### Quality assessment and risk of bias

For quality assessment of the included studies, the Mixed Methods Appraisal Tool (MMAT) was used [25]. The tool is divided into five categories with different methodological quality criteria that are used depending on the study design and methods: qualitative, quantitative (categorised into: randomised controlled, nonrandomized, and descriptive), and mixed methods. Every criterion is rated as ‘yes’, ‘no’, ‘partial’ or ‘cannot tell’ for every applicable item. The authors assessed the included studies using the MMAT tool independently and then met to compare scores. Any disagreements were discussed until consensus was reached. Methodological quality scores were as follows: 0% (no quality), 25% (low quality), 50% (moderate quality), 75% (considerable quality), and 100% (high quality).

## Results

### Search results

**Fig. 1:**
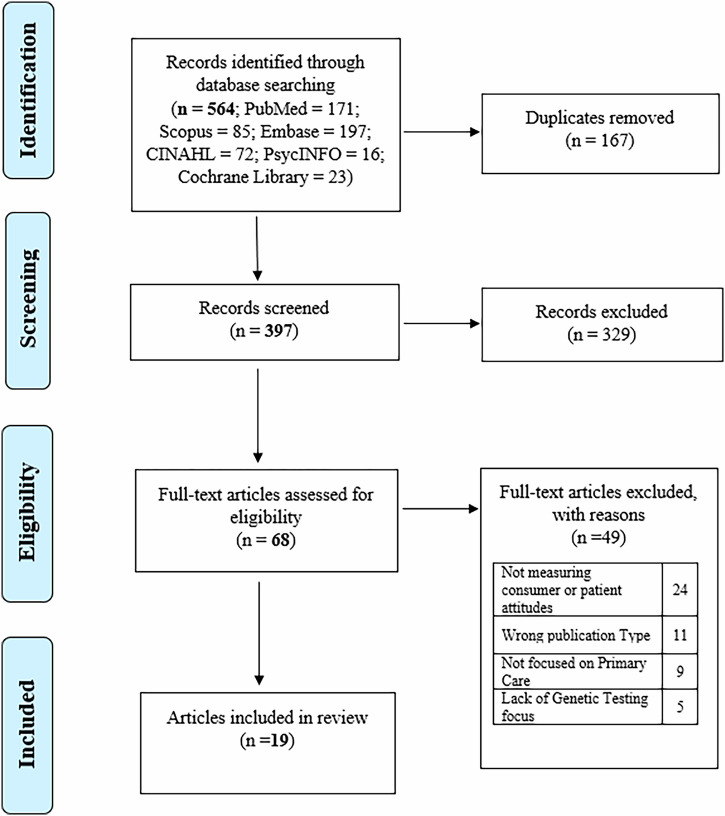
PRISMA flowchart displaying the process of identification and selection of included articles.

A total of 564 records were retrieved from the databases. Of these, 167 were duplicates and thus removed, resulting in 397 records for title and abstract screening. From these, 329 were excluded for not meeting eligibility criteria. A total of 68 full text articles were screened, of which 19 met the eligibility criteria (See Fig. 1 for PRISMA flow diagram). No additional studies were found from snowballing the reference lists of included records; therefore 19 records were included in the final review.

### Study characteristics

The main characteristics of the 19 included studies are shown in Table 2. Of these, six studies were conducted in the US [26–31], seven in Europe (Germany, Poland, The Netherlands, UK) [32–38], four in Australia [39–42] and two in Canada [43, 44].

**Table 2:** Characteristics of included studies (*n* = 19).

Author, Study Setting	Study Aim	Study Design and Methodology	Health condition/setting (family physician GP or other)	Type of Genetic Testing	Participants (n)	Sex (male/female)
Harris et al., [31], USA	To assess the acceptability of integrating CF carrier testing into antenatal care by GPs at the first booking appt.	Questionnaires, interviews	Cystic fibrosis (Primary Care)	Carrier screening	n = 75	42 women in one group and 34 (cis) couples in the other.
Leventhal et al. 2012, USA	We explored primary care patients’ interest, attitudes, and preferences related to genomic testing and receipt of test results for common complex diseases.	Focus groups	Colorectal cancer (Primary Care)	SNP testing for colorectal cancer risk.	n = 24	62% female
Armstrong et al., [27], USA	The objective of the study was to investigate the factors associated with interest in BRCA1/2 testing among primary care patients receiving only limited information about testing.	Cross sectional Survey	Breast cancer (Primary Care)	Screening for BRCA 1/2	n = 242	All female
Williams et al., [28], USA	To compare group differences in familiarity. perceptions, and preferences for precision medicine.	Cross sectional Survey	(Primary Care)	Precision and personalised medicine	n = 252	35.5%/65.1%
Miller et al., [29], USA	To explore the expectations of patients or the PCP role once genetic test results are received	Semi-structured interviews	Breast/ovarian cancer or hereditary nonpolyposis colorectal cancer (Primary Care)	Genetic testing for BRCA1, BRCA2, HNPCC	Set 1: 25 Set 2: 21	Set 1: Females 22 Males 3; Set 2 Females 19 Males 2
Saya et al., [39], Australia	To explore informed decision-making and attitudes towards genomic testing to predict personal risk of colorectal cancer (CRC)	Quantitative: survey; Qualitative: interview	Colorectal cancer (Primary Care)	Colorectal cancer genomic test to predict personal risk of colorectal cancer	Quantitative: 135 Qualitative: 16	Qualitative: n = 12/4 Quantitative: n = 46/89
Silva et al., [38], UK	To explore patient and health professional experiences of introducing genetic testing with case finding for FH in primary care	Qualitative: Semi-structured interviews	Familial hypercholesterolaemia (FH)/(General practice)	Diagnostic genetic testing for familial hypercholesterolaemia	24 PCP, 17 PC Professionals	n = 11/13
Rogausch et al., [32], Germany	To explore patients and physicians’ perspectives on the potential implications of pharmacogenetic testing, and to analyse the possible determinants of their expectations	Cross-sectional study using telephone interviews	Pharmacogenetics (Primary Care	Pharmacogenetics	Patients, n = 196; GPs, n = 106	Patients: 55% female; GPs: 25% female
Hernandez et al., [33], Germany	Generate stakeholder input for a statewide strategic plan for genetic services in the southwestern region of the United States	Semi-moderated focus groups consumers and service providers	Rare genetic diseases (Primary Care)	Genetic screening	28 consumers and 38 service providers	n = 11/55
Poppelaars et al., [34], Germany	This qualitative study aimed to explore possibilities and barriers in the implementation of a nationwide preconceptional cystic fibrosis (CF) carrier screening programme.	Focus Groups	Cystic fibrosis (General practitioners and municipal health service workers)	Cystic fibrosis carrier screening	n = 46	n = 19/27
Teixeira et al., [36], France	To explore hereditary haemochromatosis (HH) patients’ perspectives on genetic information, namely the types of sources used, preferred or trusted.	Qualitative Online survey	Haemochromatosis (Primary Care)	Genetic screening	n = 895	n = 378/517
Middlemass et al., [44], Canada	To explore how patients who have had a recent conventional cardiovascular risk assessment, perceive additional information from genetic testing for CHD	Qualitative interview study	Cardiovascular disease (Primary Care)	Genetic testing for coronary heart disease	n = 119	n = 40/69
Haga et al., [42], Australia	To investigate patient experiences with pharmacogenetic (PGx) testing	Online Survey	Pharmacogenetics (Primary Care)	Pharmacogenetics	n = 63	n = 30/43
Hay et al., [43], Canada	We examined the level of interest in pursuing MC1R testing, and patterns of interest across skin cancer perceived threat and control attitudes, cultural beliefs	Online Survey	Skin cancers (Primary Care)	Genetic screening	n = 499	Not stated
Frigon et al., [41], Australia	To better understand the perceptions of PCPs, pharmacists and patients on the implementation of pharmacogenomic testing in clinical practice	Semi-structured focus group interviews	Pharmacogenetics (Physicians, pharmacists)	Pharmacogenetics	Patients n = 30, PCP n = 23, Pharmacist (n = 11)	Patients n = 9/21, PCP n = 6/17, Pharmacists n = 2/9
Wilde et al., [40], Australia	To qualitatively assess public understanding of, and attitudes towards, risk prediction involving susceptibility genes for depression based on *5–HTTLPR* genotyping.	Focus Groups interviews	Psychiatric conditions (Primary Care)	Genetic screening	n = 36	n = 18/18
Vande Perre et al., [30], USA	To compare the expectations of the GPs’ role according to BRCA1/2 mutation carriers and to GPs themselves	Quantitative descriptive survey	Breast cancer (Primary Care)	Genetic testing (BRCA1/2)	n = 176	Not stated
Puryear et al., 2017, The Netherlands	To identify and compare patient and primary care provider (PCP) expectations of genetics services in primary care.	Mixed method: Quantitative: survey; Qualitative: interview	Multiple conditions (Primary Care)	Genetic screening	100 (quant); 20 (qual)	61% F (quant); 65% F (qual)
Helmes et al., [37], Germany	We conducted a study on women’s preferred physician involvement in the decision to obtain genetic testing for breast cancer risk.	Quantitative descriptive survey	Breast cancer (Primary Care)	Genetic testing for breast cancer risk	n = 340	100% female

*BRCA* Breast Cancer gene 1*, CF* Cystic Fibrosis*, HNPCC* Hereditary Non polyposis Colorectal Cancer, *PCP* Primary care provider, *Pgt* Personal genomic test, *qual* Qualitative, *quant”* Quantitative, *SNP* Single nucleotide polymorphism

Thirteen studies utilised qualitative methodology [26–29, 31–34, 36, 38, 40, 41, 44], including structured or semi structured focus groups [26, 29, 31, 33, 34, 40, 41]. Three used cross-sectional surveys [27, 28, 32]. Four studies were mixed methods [35, 39, 42, 43] and two were quantitative [30, 37].

### Type of genetic application

Type of genetic application varied greatly amongst the included studies, including diagnostic tests for hereditary haemochromatosis [36], Familial Hypercholesterolaemia [38], and BRCA (BReast CAncer gene) or HNPP (Hereditary Nonpolyposis Colorectal Cancer) [27, 30] screening for genetic predisposition for psychiatric conditions [40] and coronary heart disease [44], pharmacogenomics [32, 41] and reproductive carrier screening for cystic fibrosis (CF) [31, 34]. See Table 2 for details of the included studies.

### Participants

A total of 3557 participants were included across the 19 studies. The type of participants involved in these studies were diverse, with the majority being primary care consumers or patients with a specific health condition. Although PCPs’ expectations were explored across seven studies, their results were not included in the analysis [29, 30, 32, 33, 35, 38, 41]. Many participants had a pre-existing condition or a family history of a genetic condition. Most studies focused on lived experiences of genetic testing or screening. The included studies did not always provide information on the background of primary care physicians (e.g., whether they were experienced with genetic testing or not). Eighteen studies focused on participants considering genetic testing for themselves, and one study examined the experiences of parents undergoing testing for a child [33]. See Table 2 for details of the included studies.

### Quality assessment and risk of bias

All included studies were deemed as being of good quality (see Appendix 2 for MMAT appraisal). Only one of the 19 included studies had one or more issues. This study utilised a mixed methods approach. The quality issues included outputs of the integration of qualitative components being adequately interpreted and different components of the study effectively integrated to answer the research question.

### Thematic analysis

**Fig. 2:**
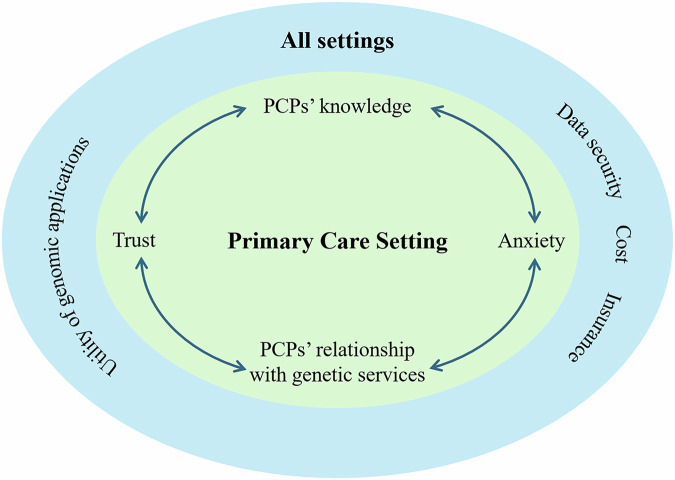
Themes from included papers Themes from the included papers: outer circle are general attitudes to genomic applications and the inner circle are specific to genomic applications in the primary care setting.

The focus of this review was consumers’ perceptions and experiences of genetic applications in the primary care setting. Most studies included an assessment of the acceptability of the genomic applications themselves, without reference to the setting. The two overarching concepts emerging from the analysis were: (1) consumer views on genomic testing that were unique to the primary care setting with four themes identified: trust in the PCP, lack of knowledge and resources for PCPs, anxiety and emotional support needs, and PCP’s relationship with genetic professionals; and (2) consumer views on genomic testing irrespective of setting, with three themes identified: beliefs about genetic applications, data security, insurance concerns and costs. These latter themes aligned with what is known from the broader body of evidence on genetic testing by genetic professionals or in hospital settings. These concepts and associated themes are explained in detail below (see also Fig. 2).

### Genomic testing in the primary care setting

#### Trust in the primary care provider

Consumers’ attitudes surrounding the trust placed in their PCP was a key influencer of a consumers’ acceptance and engagement with new medical technologies such as genetic testing [29]. This trust was built on the perception that their provider is knowledgeable, competent, and had the consumers’ best interests at heart [28]. A strong patient-provider relationship, characterised by open communication and mutual respect, was reported to enhance consumers’ confidence in medical recommendations, making them more likely to consider genetic testing as a valuable tool for personalised healthcare in primary care [28, 34]. Similarly, trust in the provider was a key determinant in consumers’ perceptions of the risks and benefits of genetic testing, reinforcing their personal decision [32]. Consumers trust in their PCP stemmed from long-standing relationships developed through years of family and individual consultations, in contrast to the genetic specialist they were meeting for the first time. Testing through their PCP was therefore seen as offering greater accessibility and convenience compared to other settings [29, 31].

#### PCPs’ Knowledge and consumer resources

Consumers in some studies were confident in their PCP’s knowledge and awareness of genetic testing and were comfortable discussing genetic test results with their provider [28, 30, 32]. For parents with children born with a genetic condition, consumers stated that communities lack sufficient knowledge about genetic conditions, meaning they faced a steep learning curve that required extensive support from PCPs [33]. Consumers mentioned that when PCPs failed to provide the information that they needed, or were unable to answer questions, they often sought it through other means such as the internet [33] (See Table 3).

**Table 3:** Themes and representative quotes illustrating participant perspectives.

Theme	Example quote from participant perspective	References
Trust in the Primary care provider	*“And if she [GP] were to see something, I would number one: I would expect she would be smart enough to see it if it were there; and two: she would mention it. And I’d be certainly willing and interested to talk with her about it… I would be open of course, but if no one brings it up I just let it go. Why should I, you know?” (*Wilde et al., [40]*)*	[28, 29, 31, 32, 34]
Primary care physician’s knowledge and consumer resources	*“Of the 1019 survey respondents, 66% would like to receive information on the disease from a family physician, but 69% agreed that the specialist was the one they trusted more.” (*Teixeira, et al. [36])	[28, 30, 32, 33]
Anxiety and emotional support	*“It takes time, it takes time to accept [genetic test result]. That’s how it was for me. I didn’t accept. When I went for my visits with Dr A., I cried all the time.”* [Mother] (Hernandez et al., 2019)	[28, 33, 38, 41]
Primary care physician’s relationship with genetic professionals	*“Most of it [seeing specialists] was questions, questions… he didn’t explain… how I am ever going to find out whether I have got this condition”* [PP5 referred to specialist as was found to have a variant of unknown significance] *(*Silva et al., [38]*)*	[29, 35, 41]
Beliefs about genomic applications	*“You get this test and it tells you everything about your risks and your genetic makeup. So I just think it could be interesting to know, you know, just again to help me keep healthy for the future” (*Puryear et al., [35]*)*	[27, 28, 31, 32, 34, 38–44]
Data Security	*“The only reason I took part in this genetic test was because it was guaranteed to be confidential and not passed on to any other party.” (*Middlemass et al., [44]*)*	[26, 27, 38, 40]
Insurance costs and concerns	*“What are the implications of this? If you know this and you got an increased risk and it goes into your medical history, life insurance companies take those medical histories into account when they are coming up with plans.” (*Leventhal et al., [26]*)*	[28, 29, 33, 35, 39]

#### Anxiety and emotional support

A recurring consumer concern in the primary care setting related to the PCPs’ ability to respond to anxiety associated with genetic testing and the receipt of results. Consumers expressed that waiting for genetic information can be anxiety-inducing, with many feeling that psychological support from their PCP was necessary both while awaiting test results and after receiving a result [38, 41] (See Table 3). Consumers stated that for children with genetic conditions, PCPs may not have sufficient knowledge or experience to emotionally support families [33]. In a study by Vande Perre et al. [30], 23.9% of consumers considered providing psychological support to be part of their PCP’s role. Additionally, counselling about test results tailored to a consumers’ health literacy and cultural background was considered very important when deciding whether to undergo genetic testing in the primary care setting [28].

#### PCPs’ relationship with genetic professionals

Consumers valued their PCP’s involvement in genetic testing and expected their PCP to discuss the possibility of testing, provide referrals to specialists (geneticists and genetic counsellors) when necessary, and incorporate genetic results into their care, even if the PCP did not initiate the tests [35]. However, while consumers wanted their PCPs to play a role in their overall care, two studies reported that consumers preferred for genetic results to be interpreted by a specialist, such as a geneticist or counsellor, due to the need for specialised expertise in genetic medicine [29, 35]. Regarding pharmacogenomic testing, consumers were divided: while many supported pharmacist involvement, some preferred that their PCP managed their care directly. In one study a significant majority (80%) of consumers preferred to be informed about genetic testing by their PCP rather than a specialist [41], although they unanimously rejected the idea of seeking genetic testing from biotechnology companies or third parties, expressing a strong preference for face-to-face consultations with their PCP over non-face-to-face genetic counselling.

### Consumer views on genomic testing irrespective of setting

#### Beliefs about genomic applications

Twelve studies discussed how consumers valued the knowledge gained from genetic testing in understanding their personal risk factors (e.g., for breast cancer [27]). This belief extended beyond the value of the individual to the value to family members, for example, the benefit genetic testing provides for dominant conditions such as Familial Hypercholesterolaemia and carrier status for CF [28, 31, 34]. Consumers considered it beneficial to explore family history and whether genetic testing was appropriate to their particular heredity [38, 39]. Other motivations for accepting genetic testing were linked to concerns about and perceptions of a specific condition. For example, in one study, the main reported motivation for participants accepting an offer of genetic testing for coronary heart disease risk was a family history that they wished to clarify, especially when they had passed the age of a relative’s death from the conditions related to coronary heart disease [44]. Growing knowledge of genetic influences on health was found to increase interest in learning about personal genetic risk for common diseases whilst also serving to promote interest and uptake of genetic testing in primary care settings [43]. The value gained from conducting pharmacogenomic testing in the primary care setting also added to consumers beliefs on genomic testing and the invaluable knowledge of personal risk factors of an individual’s genetic makeup [32, 41, 42]. Participants reported that genetic testing could help reduce the stigma associated with mental illnesses like depression by validating them if a genetic component is found [40].

#### Data security

Consumers reported concern about the confidentiality of test results, particularly regarding potential access by unauthorised individuals, such as employers, insurance providers, or third parties without their explicit consent [27, 38]. Consumers reported confidentiality of genetic information as a top priority, as breaches could lead to significant personal and financial consequences, especially relating to insurance eligibility [40]. Concerns were also raised regarding data sharing beyond a treating specialist and the incorporation of results into participants’ electronic medical records [26]. In one study, consumers commented on data security as a strong reason against having genomic testing, stating that they would not trust the confidentiality of genetic test results if obtained through the public health system [40].

#### Insurance concerns and costs

Several studies from the US reported consumers’ significant concerns about the cost of genetic testing, insurance coverage and out of pocket expenses. Despite having private health insurance, participants reported being frustrated by the financial burden imposed by the limited coverage for genetic services [33]. The upfront costs of genetic tests, ranging from a few hundred to several thousand dollars depending on complexity, were seen as prohibitive, especially when insurance did not cover the tests [28, 33]. Tests costing more than $500 were cited as a major barrier, with consumers stating they would forgo testing at such prices [35].

There were additional concerns that genetic test results might lead to insurance discrimination, with insurers potentially denying coverage, raising premiums, or excluding certain conditions based on genetic risk factors [33]. The lack of standardised insurance regulations also raised fears of employment discrimination following a genetic diagnosis [29, 39]. Consumers noted that some conditions were deemed untreatable by insurers and, therefore, not covered, leaving them unable to be reimbursed [33].

## Discussion

This systematic review examined consumers’ knowledge, attitudes, perspectives, and experiences regarding genetic testing in primary healthcare settings. Our findings reflect that consumer views on genetic tests vary by context, but with several recurring themes. Many consumers express concerns about the cost of genetic tests, questioning whether their benefits justify the expense, especially given the high costs that can hinder broader adoption in some countries. As with studies on genetic testing in clinical research and direct-to-consumer settings, our findings show that privacy and data security are also significant issues in the primary care setting. Consumers reported worrying about access to their genetic information and the potential misuse of data by third parties, such as insurance companies or employers, leading to fears of discrimination. Uniquely to the primary care context, we found that the trust established in the doctor-patient relationship could facilitate consumers’ openness to genetic testing, as they are more likely to perceive it as recommended by a trusted physician offering personalised guidance. The primary care setting also offers consumers greater convenience and a perception of greater accessibility. Additionally, uncertainty about the utility of genetic tests in primary care persists here as in other settings, with consumers questioning their relevance to their health and whether results will provide valuable insights or contribute to anxiety.

Our findings underscore that data security and concerns about costs and insurance are critical factors influencing individuals’ decisions to undergo genomic testing in primary care as reported in other settings [33, 40]. These issues often lead to hesitancy, as people fear potential financial and social repercussions. Previous studies have explored these consumer concerns regarding costs, privacy, data security, and insurance implications. For instance, Haga et al. [17] identified testing costs as a significant barrier, especially with out-of-pocket expenses [17]. Participants also expressed major worries about the potential misuse of their genetic information by third parties and the risk of genetic discrimination by insurers, which could result in higher premiums or coverage denial [45, 46]. It could be argued that consumers who engage in DTC genetic testing are less concerned with data security issues, yet for primary care consumers more broadly, data security appears to be a prevailing concern. This paradox may stem from the degree of consumers’ perceived control; DTC tests, being self-initiated, may contribute to a sense that one is in control, while clinical testing involves a more formalised process. Trust dynamics may also play a role, as processes within healthcare institutions may be perceived as opaque, whilst DTC companies market themselves as consumer friendly and transparent, despite profiting from the genetic data [47]. The lack of comprehensive protections in many countries against genetic discrimination can exacerbate anxiety associated with genomics testing, highlighting the need for robust safeguards and transparent communication from healthcare providers and policymakers. We note that recently in Australia, life insurance regulations were tightened to prevent companies from using genetic testing to refuse cover, bringing it into line with the UK and Canada [48]. Additionally, clear guidelines and legal protections, like those provided by the Genetic Information Non-discrimination Act (GINA) in the US, are crucial in addressing these concerns and fostering a more supportive environment for individuals undergoing genomic testing [49, 50].

Our results show that consumers’ views on genomic testing in the primary care setting reflect a unique interplay between the knowledge and relationship PCPs have with genetic services, and consumers’ own anxiety around genetics testing and trust in PCPs (Fig. 2). The included studies commented that consumers generally trust their PCPs to initiate discussions about genetic testing, refer them to specialists and incorporate test results into their care [35]. However, consumers undergoing genomic testing express anxiety that can arise from the potential outcomes and interpretations of this genetic information. The relationship between PCPs and genetic services is crucial in managing this anxiety as consumers often rely on their PCPs to navigate the complexities of genetic testing, while also valuing the specialised expertise of genetic counsellors and specialists for interpreting results [51].

Our results highlighted that PCPs are uniquely positioned to promote genomic testing uptake for a broader population by facilitating informed decision-making. As the first point of contact for many consumers, PCPs have established relationships built on trust, allowing them to communicate the benefits, risks, and limitations of genomic testing in a way that is accessible and personalised. Studies have reported that consumers prefer receiving pharmacogenomic test results from familiar providers whom they trust such as a PCP, rather than a pharmacist who is arguably more knowledgeable about the medications’ actions [52, 53]. This preference was consistent across both parents and consumers, with no significant differences found. This trusted role enables PCPs to guide consumers through complex information, address concerns, and ensure that decisions about testing are made in alignment with the consumers’ values and health goals. Samuel et al. (2017) argued that offering a genomic test should be viewed as an on-going collaborative decision-making process between a healthcare provider andthe consumer. This process should build trustworthiness, openness, and honesty while considering the consumer’s autonomy and privacy, and as such can be seen as inextricably linked with ethical considerations [54].

For parents of children with suspected genetic conditions, inaccurate or incomplete information provided to parents can undermine their trust in genetic testing, leading to anxiety or incorrect assumptions about their child’s health [33]. Certain testing such as for psychiatric predisposition is typically not offered in primary care due to its complexity and availability in this setting. These tests involve intricate genetic information that may be difficult for GPs to interpret accurately, posing a risk of misdiagnosis or misinformation. This can result in emotional distress or misguided decisions, especially when there is a risk of inadvertent disclosure or misinterpretation of test results, ultimately affecting consumers’ perceptions of the reliability and value of such testing [55]. The risk of inadvertent disclosure or misinterpretation of results may further impact consumers’ trust in the reliability and value of such testing [55].

Appropriate use of genomic testing in the primary care setting emerged as a concern for consumers because PCPs do not have the same level of expertise in genetics as specialised genetic professionals [56]. PCPs often receive limited genetics training as part of their medical education, and because genetics is a constantly and rapidly evolving field, contact and collaboration with genetics providers is needed to bridge the knowledge gap [56, 57]. A key question is whether differences in knowledge prevail between consumers with little exposure to genetics (genetics-naïve) and those with a firsthand experience or prior knowledge (genetics-informed). The former may overestimate PCP expertise due to general trust in their healthcare provider, while the latter may have higher expectations based on direct engagement with specialists and prior knowledge. For PCPs who lack both knowledge regarding genetic testing and access to guidance on appropriate testing, they may have to seek assistance from other professionals or resort to other options, including independently ordering testing without guidance [57]. This lack of support can influence both consumer perceptions of their PCP’s ability to offer testing appropriately and manage the associated anxiety and quality of care delivered to consumer, if PCP do not have guidance. In the primary care setting, a 2017 study showed that PCPs express apprehension about learning concepts and language surrounding genomics and whole genome sequencing and report preparing extensively before disclosing results to consumers by using educational resources with which they are familiar [58]. Previous studies have documented that genetic testing is a stressful experience for consumers [59, 60]. Generally, consumers report elevated anxiety upon receiving test results, which is unsurprising as results may have lifelong physical, social and psychological effects on the affected individual and their family [61]. Anxiety levels thus might be higher in the primary care setting compared to a genetic professional setting due to a perceived lack of relevant expertise in an PCP [62].

We found that, overall, consumers reported being satisfied with the amount of information provided by their PCP regarding genomic testing. From our findings, there was no clear evidence that certain genetic tests are more acceptable to consumers than others. However, simpler diagnostic genetic tests such as single gene diagnostic testing or small panel genetic carrier screening may be more suitable for GPs, particularly when clear guidelines and decision-making frameworks are available and established. The feasibility of offering more complex tests in primary care likely depends in part on the level of support, training and resources provided to GPs to ensure accurate interpretation and consumer counselling. In a more recent study (2022), it was noted that PCPs frequently offer information about the risks, benefits, limitations, and implications of genomic testing, though there is room for improvement in these processes [63]. Implementation strategies involving training and education for healthcare providers and greater informational support for consumers to fill the knowledge gap and improve consistency of recommendations and guidelines of genomic testing are key to supporting routine testing in primary care [63]. Successful education strategies are critical to determining the outcome of genomic programmes and should be embedded in key steps of implementation [64, 65].

Future research to boost consumer perceived knowledge and increase consumer-provider trust should focus on ensuring that genomic testing benefits have been clearly explained and understood, specifically in areas that require a heightened degree of specialty such as reproductive carrier screening [66]. The Australian Reproductive Genetic Carrier Screening project, for example included a decision support tool for couples planning for pregnancy or in early pregnancy to decide if the test aligned with their values and was appropriate for them. It provided education on reproductive carrier screening including the test’s limitations and the consequences of a high-chance result [9]. Other specialties such as obstetrics and gynaecology, and oncology have begun to integrate genetic testing and referrals into daily practice [67]. The benefits in primary care are not yet demonstrated but this review demonstrates that there are many opportunities and pitfalls to navigate. Genetic professionals should continue to work with PCPs and consumers to incorporate genetic services into primary care settings and bridge the gap between their expectations and practice.

### Strengths and limitations

The strengths of this systematic review include its broad search strategy which aimed to capture all published literature on consumer experiences associated with genetic testing. Firstly, a limitation of this review is the inclusion of studies from diverse healthcare systems worldwide, where the role of PCPs in genomics may vary based on system-specific policies, resources, and professional expectations. These differences could influence findings on PCP engagement, consumer expectations, and the feasibility of integrating genomic medicine into primary care. While we aimed to synthesise common themes, variations across healthcare settings should be considered when interpreting the results. Secondly, while comprehensive, we acknowledge that some nuanced terminology related to specific PCP roles may not have been fully captured by the search strategy meaning some studies may have been missed. Thirdly, the articles included in this systematic review span a wide date range; however, despite genetics being a fast-moving field, the factors identified remain relevant to current research and were analysed. Finally, we did not include studies utilising DTC testing. We acknowledge that this method impacts PCPs even though they did not order the tests. These tests therefore may impact consumer attitudes which warrant further research.

## Conclusion

We have identified key themes associated with consumers’ knowledge, attitudes and experiences undergoing genetic testing in primary care. Understanding these attitudes in genetic testing may be useful in leveraging beneficial health outcomes in primary care and broader healthcare settings. Recognising that the benefit of genomic applications, as well as concerns associated with data security, costs and insurance, play a key role in consumer attitudes is critical to improving uptake and experiences of genetics testing in primary care. Factoring in the importance of PCP knowledge, available resources and skills to manage consumer anxiety will be essential in facilitating effective genomic medicine practices in primary care, leading to greater targeted interventions and improved patient care.

## Supplementary information

Supplementary 1

Supplementary 2

Supplementary 3

## Data Availability

The datasets used and/or analysed during the current study are available from the corresponding author on reasonable request.

## References

[1] Best S, Long JC, Gaff C, Braithwaite J, Taylor N. Investigating the adoption of clinical genomics in Australia. An implementation science case study. Genes. 2021;12:317. 33672413 10.3390/genes12020317 PMC7926693

[2] Eckstein L, Chalmers D, Critchley C, Jeanneret R, McWhirter R, Nielsen J, et al. Australia: regulating genomic data sharing to promote public trust. Hum Genet. 2018;137:583. 30116956 10.1007/s00439-018-1914-z PMC6132638

[3] Paltiel M, Taylor M, Newson A. Protection of genomic data and the Australian Privacy Act: when are genomic data ‘personal information’?. Int Data Priv Law. 2023;13:47–62.

[4] Shi L. The impact of primary care: a focused review. Scientifica. 2012;2012:432892. 24278694 10.6064/2012/432892 PMC3820521

[5] Kringos D, Boerma W, Bourgueil Y, Cartier T, Dedeu T, Hasvold T, et al. The strength of primary care in Europe: an international comparative study. Br J Gen Pr. 2013;63:e742–50. 10.3399/bjgp13X674422 PMC3809427 24267857

[6] European Association for the Study of the Liver. EASL clinical practice guidelines for HFE hemochromatosis. J Hepatol. 2010;53:3–22. 20471131 10.1016/j.jhep.2010.03.001

[7] Practitioners TRAC of general. Australian Family Physician. The Royal Australian College of General Practitioners; [cited 2025 Mar 17]. Detecting familial hypercholesterolaemia in general practice. Available from: https://www.racgp.org.au/afp/2012/december/familial-hypercholesterolaemia.

[8] Arishi WA, Alhadrami HA, Zourob M. Techniques for the detection of sickle cell disease: a review. Micromachines. 2021;12:519. 34063111 10.3390/mi12050519 PMC8148117

[9] Archibald AD, McClaren BJ, Caruana J, Tutty E, King EA, Halliday JL, et al. The Australian Reproductive Genetic Carrier Screening Project (Mackenzie’s Mission): Design and Implementation. J Pers Med. 2022;12:1781. 36579509 10.3390/jpm12111781 PMC9698511

[10] Foo D, Spanos S, Dammery G, Ellis LA, Willcock SM, Braithwaite J. The rise of direct-to-consumer telemedicine services in Australia: implications for primary care and future research. Med J Aust. 2023;Online first. Available from: https://www.mja.com.au/journal/2023/219/8/rise-direct-consumer-telemedicine-services-australia-implications-primary-care. 10.5694/mja2.52097 37666506

[11] Cormack M, Irving KB, Cunningham F, Fennell AP. Mainstreaming genomic testing: pre-test counselling and informed consent. Med J Aust. 2024;Online first. Available from: https://www.mja.com.au/journal/2024/220/8/mainstreaming-genomic-testing-pre-test-counselling-and-informed-consent. 10.5694/mja2.52254 38479398

[12] Long JC, Gaff C, Clay C. Transforming the genomics workforce to sustain high value care [Internet]. Deeble Institute for Health Policy Research; 2022. Available from: https://apo.org.au/node/317075https://apo.org.au/sites/default/files/resource-files/2022-03/apo-nid317075.pdf.

[13] Harding B, Webber C, Ruhland L, Dalgarno N, Armour CM, Birtwhistle R, et al. Primary care providers’ lived experiences of genetics in practice. J Commun Genet. 2019;10:85–93. 10.1007/s12687-018-0364-6 PMC6325046 29700759

[14] Pearce A, Mitchell LA, Best S, Young MA, Terrill B. Publics’ knowledge of, attitude to and motivation towards health-related genomics: a scoping review. Eur J Hum Genet. 2024;32:747–58. 38316954 10.1038/s41431-024-01547-5 PMC11220043

[15] Wallingford CK, Cutler K, Istiko SN, Fowles LF, Lamb R, Bean J, et al. Queensland consumers’ awareness and understanding of clinical genetics services. Front Genet. 2020;11:537743. 33193608 10.3389/fgene.2020.537743 PMC7593610

[16] Likhanov M, Zakharov I, Awofala A, Ogundele O, Selita F, Kovas Y, et al. Attitudes towards genetic testing: The role of genetic literacy, motivated cognition, and socio-demographic characteristics. PLoS ONE. 2023;18:e0293187. 37967060 10.1371/journal.pone.0293187 PMC10651000

[17] Haga SB, Barry WT, Mills R, Ginsburg GS, Svetkey L, Sullivan J, et al. Public knowledge of and attitudes toward genetics and genetic testing. Genet Test Mol Biomark. 2013;17:327–35. 10.1089/gtmb.2012.0350 PMC3609633 23406207

[18] Cumpston M, Li T, Page MJ, Chandler J, Welch VA, Higgins JP, et al. Updated guidance for trusted systematic reviews: a new edition of the Cochrane Handbook for Systematic Reviews of Interventions. Cochrane Database Syst Rev. 2019;2019:ED000142. 10.1002/14651858.ED000142 PMC10284251 31643080

[19] Page MJ, McKenzie JE, Bossuyt PM, Boutron I, Hoffmann TC, Mulrow CD, et al. The PRISMA 2020 statement: an updated guideline for reporting systematic reviews. BMJ. 2021;372:n71. 33782057 10.1136/bmj.n71 PMC8005924

[20] Ouzzani M, Hammady H, Fedorowicz Z, Elmagarmid A. Rayyan—a web and mobile app for systematic reviews. Syst Rev. 2016;5:210. 27919275 10.1186/s13643-016-0384-4 PMC5139140

[21] Ruhl GL, Hazel JW, Clayton EW, Malin BA. Public attitudes toward direct to consumer genetic testing. AMIA Annu Symp Proc. 2020;2019:774–83. 32308873 PMC7153088

[22] Braun V, Clarke V. Using thematic analysis in psychology. Qual Res Psychol. 2006;3:77–101.

[23] Hong QN, Pluye P, Bujold M, Wassef M. Convergent and sequential synthesis designs: implications for conducting and reporting systematic reviews of qualitative and quantitative evidence. Syst Rev. 2017;6:61. 28335799 10.1186/s13643-017-0454-2 PMC5364694

[24] Sandelowski M. Telling stories: narrative approaches in qualitative research. Image J Nurs Sch. 1991;23:161–6. 1916857 10.1111/j.1547-5069.1991.tb00662.x

[25] Hong QN, Fàbregues S, Bartlett G, Boardman F, Cargo M, Dagenais P, et al. The Mixed Methods Appraisal Tool (MMAT) version 2018 for information professionals and researchers. Educ Inf. 2018;34:285–91.

[26] Leventhal K, Tuong W, Peshkin BN, Salehizadeh Y, Fishman MB, Eggly S, et al. Is it Really Worth it to Get Tested?”: Primary Care Patients’ Impressions of Predictive SNP Testing for Colon Cancer. J Genet Couns. 2013;22:138–51. 22911325 10.1007/s10897-012-9530-x PMC3567438

[27] Armstrong K, Weber B, Ubel PA, Guerra C, Schwartz JS. Interest in BRCA1/2 Testing in a primary care population. Prev Med. 2002;34:590–5. 12052018 10.1006/pmed.2002.1022

[28] Williams JR, Yeh VM, Bruce MA, Szetela C, Ukoli F, Wilkins CH, et al. Precision Medicine: Familiarity, perceived health drivers, and genetic testing considerations across health literacy levels in a diverse sample. J Genet Couns. 2019;28:59–69. 10.1007/s10897-018-0291-z PMC6374217 30105426

[29] Miller FA, Carroll JC, Wilson BJ, Bytautas JP, Allanson J, Cappelli M, et al. The primary care physician role in cancer genetics: a qualitative study of patient experience. Fam Pr. 2010;27:563–9. 10.1093/fampra/cmq035 20534792

[30] Vande Perre P, Toledano D, Corsini C, Escriba E, Laporte M, Bertet H, et al. Role of the general practitioner in the care of *BRCA1* and *BRCA2* mutation carriers: General practitioner and patient perspectives. Mol Genet Genom Med. 2018;6:957–65. 10.1002/mgg3.464 PMC6305637 30308700

[31] Harris H, Scotcher D, Hartley N, Wallace A, Craufurd D, Harris R. Pilot study of the acceptability of cystic fibrosis carrier testing during routine antenatal consultations in general practice. Br J Gen Pr. 1996;46:225–7. PMC1239605 8703524

[32] Rogausch A, Prause D, Schallenberg A, Brockmöller J, Himmel W. Patients’ and physicians’ perspectives on pharmacogenetic testing. Pharmacogenomics. 2006;7:49–59. 16354124 10.2217/14622416.7.1.49

[33] Hernandez VR, Selber K, Tijerina MS. Visioning family-centered care in genetics: what parents and providers have to say. J Genet Couns. 2006;15:349–60. 16967330 10.1007/s10897-006-9032-9

[34] Poppelaars FAM, Van Der Wal G, Braspenning JCC, Cornel MC, Henneman L, Langendam MW, et al. Possibilities and barriers in the implementation of a preconceptional screening programme for cystic fibrosis carriers: a focus group study. Public Health. 2003;117:396–403. 14522154 10.1016/S0033-3506(03)00136-7

[35] Puryear L, Downs N, Nevedal A, Lewis ET, Ormond KE, Bregendahl M, et al. Patient and provider perspectives on the development of personalized medicine: a mixed-methods approach. J Commun Genet. 2018;9:283–91. 10.1007/s12687-017-0349-x PMC6002302 29280052

[36] Teixeira E, Borlido-Santos J, Brissot P, Butzeck B, Courtois F, Evans RW, et al. The importance of the general practitioner as an information source for patients with hereditary haemochromatosis. Patient Educ Couns. 2014;96:86–92. 24857332 10.1016/j.pec.2014.04.017

[37] Helmes AW, Bowen DJ, Bengel J. Patient preferences of decision-making in the context of genetic testing for breast cancer risk. Genet Med. 2002;4:150–7. 12180150 10.1097/00125817-200205000-00009

[38] Silva L, Condon L, Qureshi N, Dutton B, Weng S, Kai J. Introducing genetic testing with case finding for familial hypercholesterolaemia in primary care: qualitative study of patient and health professional experience. Br J Gen Pr. 2022;72:e519–27. 10.3399/BJGP.2021.0558 PMC9208733 35697509

[39] Saya S, McIntosh JG, Winship IM, Milton S, Clendenning M, Kyriakides M, et al. Informed choice and attitudes regarding a genomic test to predict risk of colorectal cancer in general practice. Patient Educ Couns. 2022;105:987–95. 34400040 10.1016/j.pec.2021.08.008

[40] Wilde A, Meiser B, Mitchell PB, Schofield PR. Public interest in predictive genetic testing, including direct-to-consumer testing, for susceptibility to major depression: preliminary findings. Eur J Hum Genet. 2010;18:47–51. 19690586 10.1038/ejhg.2009.138 PMC2987161

[41] Frigon MP, Blackburn MÈ, Dubois-Bouchard C, Gagnon AL, Tardif S, Tremblay K. Pharmacogenetic testing in primary care practice: opinions of physicians, pharmacists and patients. Pharmacogenomics. 2019;20:589–98. 31190623 10.2217/pgs-2019-0004

[42] Haga SB, Mills R, Moaddeb J, Allen Lapointe N, Cho A, Ginsburg GS. Patient experiences with pharmacogenetic testing in a primary care setting. Pharmacogenomics. 2016;17:1629–36. 27648637 10.2217/pgs-2016-0077 PMC5558503

[43] Hay JL, Meyer White K, Sussman A, Kaphingst K, Guest D, Schofield E, et al. Psychosocial and cultural determinants of interest and uptake of skin cancer genetic testing in diverse primary care. Public Health Genom. 2019;22:58–68. 10.1159/000501985 PMC7814986 31437847

[44] Middlemass JB, Yazdani MF, Kai J, Standen PJ, Qureshi N. Introducing genetic testing for cardiovascular disease in primary care: a qualitative study. Br J Gen Pr. 2014;64:e282–9. 10.3399/bjgp14X679714 PMC4001138 24771842

[45] Tiller J, Morris S, Rice T, Barter K, Riaz M, Keogh L, et al. Genetic discrimination by Australian insurance companies: a survey of consumer experiences. Eur J Hum Genet. 2020;28:108–13. 31281182 10.1038/s41431-019-0426-1 PMC6906286

[46] Kaufman DJ, Bollinger JM, Dvoskin RL, Scott JA. Risky Business: risk perception and the use of medical services among customers of dtc personal genetic testing. J Genet Couns. 2012;21:413–22. 22278220 10.1007/s10897-012-9483-0

[47] Hendricks-Sturrup RM, Lu CY. Direct-to-consumer genetic testing data privacy: key concerns and recommendations based on consumer perspectives. J Pers Med. 2019;9:25. 31075859 10.3390/jpm9020025 PMC6616921

[48] Monash University. Medicine, Nursing and Health Sciences. 2024 [cited 2024 Oct 11]. Australian Government bans genetic discrimination in life insurance: A big win for preventive health. Available from: https://www.monash.edu/medicine/news/latest/2024-articles/australian-government-bans-genetic-discrimination-in-life-insurance-a-big-win-for-preventive-health.

[49] Feldman EA. The Genetic Information Nondiscrimination Act (GINA): Public policy and medical practice in the age of personalized medicine. J Gen Intern Med. 2012;27:743–6. 22314637 10.1007/s11606-012-1988-6 PMC3358381

[50] Prince AER, Roche MI. Genetic information, non-discrimination, and privacy protections in genetic counseling practice. J Genet Couns. 2014;23:891–902. 25063358 10.1007/s10897-014-9743-2 PMC4233176

[51] Seibel E, Gunn G, Ali N, Jordan E, Kenneson A. Primary Care Providers’ use of genetic services in the southeast united states: barriers, facilitators, and strategies. J Prim Care Commun Health. 2022;13:215013192211347. 10.1177/21501319221134752 PMC9647281 36345220

[52] Fargher EA, Newman W, Qasim F, Elliott RA, Payne K. Patients” and healthcare professionals” views on pharmacogenetic testing and its future delivery in the NHS. Pharmacogenomics. 2007;8:1511–9. 18034616 10.2217/14622416.8.11.1511

[53] Payne K, Fargher EA, Roberts SA, Tricker K, Elliott RA, Ratcliffe J, et al. Valuing pharmacogenetic testing services: A comparison of patients’ and health care professionals’ preferences. Value Health. 2011;14:121–34. 21211494 10.1016/j.jval.2010.10.007

[54] Samuel GN, Dheensa S, Farsides B, Fenwick A, Lucassen A. Healthcare professionals’ and patients’ perspectives on consent to clinical genetic testing: moving towards a more relational approach. BMC Med Ethics. 2017;18:47. 28789658 10.1186/s12910-017-0207-8 PMC5549302

[55] Araia MH, Wilson BJ, Chakraborty P, Gall K, Honeywell C, Milburn J, et al. Factors associated with knowledge of and satisfaction with newborn screening education: a survey of mothers. Genet Med. 2012;14:963–70. 22899093 10.1038/gim.2012.87 PMC3908555

[56] Chou AF, Duncan AR, Hallford G, Kelley DM, Dean LW. Barriers and strategies to integrate medical genetics and primary care in underserved populations: a scoping review. J Commun Genet. 2021;12:291. 10.1007/s12687-021-00508-5 PMC7849219 33523369

[57] Mikat-Stevens NA, Larson IA, Tarini BA. Primary-care providers’ perceived barriers to integration of genetics services: a systematic review of the literature. Genet Med. 2015;17:169–76. 25210938 10.1038/gim.2014.101

[58] Christensen KD, Vassy JL, Jamal L, Lehmann LS, Slashinski MJ, Perry DL, et al. Are physicians prepared for whole genome sequencing? a qualitative analysis. Clin Genet. 2016;89:228–34. 26080898 10.1111/cge.12626 PMC4683111

[59] McConkie-Rosell A, Spiridigliozzi GA, Rounds K, Dawson DV, Sullivan JA, Burgess D, et al. Parental attitudes regarding carrier testing in children at risk for fragile X syndrome. Am J Med Genet. 1999;82:206–11. 10215541

[60] Pilnick A, Dingwall R. Research directions in genetic counselling: a review of the literature. Patient Educ Couns. 2001;44:95–105. 11479050 10.1016/s0738-3991(00)00181-6

[61] Dinc L, Terzioglu F. The psychological impact of genetic testing on parents. J Clin Nurs. 2006;15:45–51. 16390523 10.1111/j.1365-2702.2005.01228.x

[62] Harding B, Webber C, Rühland L, Dalgarno N, Armour C, Birtwhistle R, et al. Bridging the gap in genetics: a progressive model for primary to specialist care. BMC Med Educ. 2019;19:195. 31185964 10.1186/s12909-019-1622-y PMC6558677

[63] Dusic EJ, Theoryn T, Wang C, Swisher EM, Bowen DJ. Barriers, interventions, and recommendations: Improving the genetic testing landscape. Front Digit Health. 2022;4:961128. 36386046 10.3389/fdgth.2022.961128 PMC9665160

[64] Clyman JC, Nazir F, Tarolli S, Black E, Lombardi RQ, Higgins JJ. The impact of a genetics education program on physicians’ knowledge and genetic counseling referral patterns. Med Teach. 2007;29:e143–50. 17978961 10.1080/01421590701477373

[65] Hauser D, Obeng AO, Fei K, Ramos MA, Horowitz CR. Views of primary care providers on testing patients for genetic risks for common chronic diseases. Health Aff Proj Hope. 2018;37:793. 10.1377/hlthaff.2017.1548 PMC6503526 29733703

[66] Best S, Long JC, Fehlberg Z, Archibald AD, Braithwaite J. Supporting healthcare professionals to offer reproductive genetic carrier screening: a behaviour change theory approach. Aust J Prim Health. 2023;29:480–9. 37156638 10.1071/PY23022

[67] O’Shea R, Taylor N, Crook A, Jacobs C, Jung Kang Y, Lewis S, et al. Health system interventions to integrate genetic testing in routine oncology services: a systematic review. PLoS ONE. 2021;16:e0250379. 34010335 10.1371/journal.pone.0250379 PMC8133413

