# Supplementary material for: A systematic review of consumers’ knowledge, attitudes and experiences of primary health professionals’ role in genomic medicine
Source: Eur J Hum Genet. 2025 Jul 2;33(12):1567–78. doi: 10.1038/s41431-025-01904-y (PMC12669570; doi:10.1038/s41431-025-01904-y)
Supplement: Supplementary file 2 — Supplementary 2 [file 41431_2025_1904_MOESM2_ESM.docx]

**MeSH terms & subject headings in RED.**

**PubMed**

| **Criteria** | **NO** | **Search terms** | **Citations** |
| --- | --- | --- | --- |
| **Attitudes** | #1 | Title/Abstract: attitud* OR belief* OR behav* OR experienc* OR knowledg* OR perception* OR perspective* OR aware*  OR Health Knowledge, Attitudes, Practice |  |
|  |  | AND |  |
| **Tests** | #2 | Title/Abstract: “genetic screen*” OR “cascade screen*” OR “genomic screen*” OR “genomic sequenc*”  OR Genetic Testing |  |
|  |  | AND |  |
| **Consumers** | **#**3 | Title/Abstract: consumer* OR patient* |  |
|  |  | AND |  |
| **Primary care** | **#4** | Title/Abstract: “general practi*” OR “family practi*”  OR Primary health care/ OR General practice |  |
|  |  | NOT |  |
|  | **#5** | Title/Abstract: “tumor test*” OR “tumour test*” OR “direct-to-consumer” |  |
| **Filters** |  | AND **English language**  **AND Human studies** | **171** |

((((attitud*[Title/Abstract] OR belief*[Title/Abstract] OR behav*[Title/Abstract] OR experienc*[Title/Abstract] OR knowledg*[Title/Abstract] OR perception*[Title/Abstract] OR perspective*[Title/Abstract]) OR (health knowledge, attitudes, practice[MeSH Terms])) AND (("genetic screen*"[Title/Abstract] OR "cascade screen*"[Title/Abstract] OR "genomic screen*"[Title/Abstract] OR "genomic sequenc*"[Title/Abstract]) OR (genetic testing[MeSH Terms]))) AND (consumer*[Title/Abstract] OR patient*[Title/Abstract])) AND ((primary health care[MeSH Terms]) OR ("general practi*"[Title/Abstract]) OR ("family practi*"[Title/Abstract]) OR (general practice[MeSH Terms])) AND ((humans[Filter]) AND (english[Filter]))

= 171

**Scopus**

| **Criteria** | **NO** | **Search terms** | **Citations** |
| --- | --- | --- | --- |
| **Attitudes** | #1 | Title/Abstract/Keywords: attitud* OR belief* OR behav* OR experienc* OR knowledg* OR perception* OR perspective* OR aware* |  |
|  |  | AND |  |
| **Tests** | #2 | Title/Abstract/Keywords: “genetic screen*” OR “cascade screen*” OR “genomic screen*” OR “genomic sequenc* OR “genetic test*” | \|  \| \| --- \| |
|  |  | AND |  |
| **Consumers** | #3 | Title: consumer* OR patient* |  |
|  |  | AND |  |
| **Primary care** | #4 | Title/Abstract/Keywords: “general practi*" OR "primary care" OR "primary health care" OR “family practi*” |  |
|  |  | NOT |  |
|  | #5 | Title/Abstract/Keywords: “tumor test*” OR “tumour test*” OR “direct-to-consumer” |  |
| **Filters** |  | AND **English language**  **AND Human studies** | **85** |

( ( TITLE-ABS-KEY ( attitud* OR belief* OR behav* OR experienc* OR knowledg* OR perception* OR perspective* OR aware* ) ) AND ( TITLE-ABS-KEY ( "genetic screen*" OR "cascade screen*" OR "genomic screen*" OR "genomic sequenc*" OR "genetic test*" ) ) AND ( TITLE ( consumer* OR patient* ) ) AND ( TITLE-ABS-KEY ( "general practi*" OR "primary care" OR "primary health care" OR "family practi*" ) ) ) AND NOT ( TITLE-ABS-KEY ( "tumor test*" OR "tumour test*" OR "direct-to-consumer" ) ) AND ( LIMIT-TO ( LANGUAGE , "English" ) ) AND ( LIMIT-TO ( EXACTKEYWORD , "Human" ) )

= 85

**EMBASE**

| **Criteria** | **NO** | **Search terms** | **Citations** |
| --- | --- | --- | --- |
| **Attitudes** | #1 | Title/Abstract: attitud* OR belief* OR behav* OR experienc* OR knowledg* OR perception* OR perspective* OR aware*  OR Patient Attitude/ |  |
|  |  | AND |  |
| **Tests** | #2 | Title/Abstract: “genetic test*” OR “cascade screen*” OR “genomic screen*” OR “genomic sequenc*” OR “genomic test*” OR Genetic Screening/ |  |
|  |  | AND |  |
| **Consumers** | **#**3 | Title/Abstract: consumer* OR patient* |  |
| **Primary care** | **#**4 | Title/Abstract: “general practi*” OR “family practi*”  OR Primary health care/ OR General practice/ |  |
| **NOT** | **#**5 | Title/Abstract: “tumor test*” OR “tumour test*” OR “direct-to-consumer” |  |
|  | **#**7 | AND **English language**  **AND Human studies** | **197** |

<https://simsrad.net.ocs.mq.edu.au/login?url=http://ovidsp.ovid.com/ovidweb.cgi?T=JS&NEWS=N&PAGE=main&SHAREDSEARCHID=18PTbqA3gLWFiQU6XgGq9dyMeObqzR1vdFs2UacKTh9yM884JZJBuzqB7K3pn3BVz>

= 197

**CINAHL**

| **Criteria** | **NO** | **Search terms** | **Citations** |
| --- | --- | --- | --- |
| **Attitudes** | #1 | Abstract/Title: attitud* OR belief* OR behav* OR experienc* OR knowledg* OR perception* OR perspective* OR aware*  OR MH “Patient Attitudes” |  |
|  |  | AND |  |
| **Tests** | #2 | Abstract/Title: “genetic test*” OR “cascade screen*” OR “genomic screen*” OR “genomic sequenc*” OR MH “Genetic Screening” | \|  \|  \| \| --- \| --- \| |
|  |  | AND |  |
| **Consumers** | **#**3 | Abstract/Title: consumer* OR patient* |  |
| **Primary care** | **#**4 | Abstract/Title: “general practi*” OR  MH "Primary Health Care" OR MH "Family Practice" |  |
| **NOT** | **#**5 | Abstract/Title: “tumor test*” OR “tumour test*” OR “direct-to-consumer” |  |
|  | **#6** | AND English language | **72** |

https://search.ebscohost.com/login.aspx?direct=true&AuthType=sso&db=ccm&bquery=((((AB+(attitud*+OR+belief*+OR+behav*+OR+experienc*+OR+knowledg*+OR+perception*+OR+perspective*+OR+aware*))+OR+(TI+(attitud*+OR+belief*+OR+behav*+OR+experienc*+OR+knowledg*+OR+perception*+OR+perspective*+OR+aware*)))+OR+((MH+%26quot%3bPatient+Attitudes%26quot%3b)))+AND+(((AB+(%26quot%3bgenetic+test*%26quot%3b+OR+%26quot%3bcascade+screen*%26quot%3b+OR+%26quot%3bgenomic+screen*%26quot%3b+OR+%26quot%3bgenomic+sequenc*%26quot%3b))+OR+(TI+(%26quot%3bgenetic+test*%26quot%3b+OR+%26quot%3bcascade+screen*%26quot%3b+OR+%26quot%3bgenomic+screen*%26quot%3b+OR+%26quot%3bgenomic+sequenc*%26quot%3b)))+OR+((MH+%26quot%3bGenetic+Screening%26quot%3b)))+AND+(((AB+(consumer*+OR+patient*))+OR+(TI+(consumer*+OR+patient*)))+OR+((MH+%26quot%3bPatients%26quot%3b)))+AND+(((AB+%26quot%3bgeneral+practi*%26quot%3b)+OR+(TI+%26quot%3bgeneral+practi*%26quot%3b))+OR+((MH+%26quot%3bPrimary+Health+Care%26quot%3b)+OR+(MH+%26quot%3bFamily+Practice%26quot%3b))))+NOT+((AB+(%26quot%3btumor+test*%26quot%3b+OR+%26quot%3btumour+test*%26quot%3b+OR+%26quot%3bdirect-to-consumer%26quot%3b))+OR+(TI+(%26quot%3btumor+test*%26quot%3b+OR+%26quot%3btumour+test*%26quot%3b+OR+%26quot%3bdirect-to-consumer%26quot%3b)))&type=1&searchMode=Standard&site=ehost-live&custid=s8434881

= 72 (NOTE: no option to select “human studies”)

AB ( attitud* OR belief* OR behav* OR experienc* OR knowledg* OR perception* OR perspective* OR aware* ) OR TI ( attitud* OR belief* OR behav* OR experienc* OR knowledg* OR perception* OR perspective* OR aware* ) OR (MH "Patient Attitudes")

AND

AB ( “genetic test*” OR “cascade screen*” OR “genomic screen*” OR “genomic sequenc*” ) OR TI ( “genetic test*” OR “cascade screen*” OR “genomic screen*” OR “genomic sequenc*” ) OR (MH "Genetic Screening")

AND

AB ( consumer* OR patient* ) OR TI ( consumer* OR patient* ) OR (MH "Patients")

AND

AB “general practi*” OR TI “general practi*” OR (MH "Primary Health Care") OR (MH "Family Practice")

NOT

AB ( “tumor test*” OR “tumour test*” OR “direct-to-consumer” ) OR TI ( “tumor test*” OR “tumour test*” OR “direct-to-consumer” )

**psycINFO**

| **Criteria** | **NO** | **Search terms** | **Citations** |
| --- | --- | --- | --- |
| **Attitudes** | #1 | Title/Abstract: attitud* OR belief* OR behav* OR experienc* OR knowledg* OR perception* OR perspective* OR aware*  OR Client Attitudes/ |  |
|  |  | AND |  |
| **Tests** | #2 | Title/Abstract: “genetic screen*” OR “cascade screen*” OR “genomic screen*” OR “genomic sequenc*” OR “genomic test*” OR Genetic Testing/ |  |
|  |  | AND |  |
| **Consumers** | **#**3 | Title/Abstract: consumer* OR patient* |  |
| **Primary care** | **#**4 | Title/Abstract: “general practi*” OR “family practi*”  OR Primary health care/ OR General Practitioners/ OR Family Physicians/ |  |
| **NOT** | **#**5 | Title/Abstract: “tumor test*” OR “tumour test*” OR “direct-to-consumer” |  |
|  | **#**7 | AND **English language**  **AND Human studies** | **16** |

<https://simsrad.net.ocs.mq.edu.au/login?url=http://ovidsp.ovid.com/ovidweb.cgi?T=JS&NEWS=N&PAGE=main&SHAREDSEARCHID=3RBDkezOuHiyhjzDrER5aSTxIBRfR1oZgHYKeSDN3IrhQOtfGe2QxWQIMxJno9ZY7>

**Cochrane Library**

| **Criteria** | **NO** | **Search terms** | **Citations** |
| --- | --- | --- | --- |
| **Attitudes** | #1 | Title/Abstract/Keyword: attitud* OR belief* OR behav* OR experienc* OR knowledg* OR perception* OR perspective* OR aware*  OR Attitude to Health |  |
|  |  | AND |  |
| **Tests** | #2 | Title/Abstract/Keyword: “genetic screen*” OR “cascade screen*” OR “genomic screen*” OR “genomic sequenc*” OR “genomic test*” OR Genetic Testing/ exp |  |
|  |  | AND |  |
| **Consumers** | **#**3 | Title/Abstract/Keyword: consumer* OR patient* |  |
| **Primary care** | **#**4 | Title/Abstract/Keyword: “general practi*” OR “family practi*”  OR Primary health care/ OR General Practice/ |  |
| **NOT** | **#**5 | Title/Abstract: “tumor test*” OR “tumour test*” OR “direct-to-consumer” |  |
|  | **#**7 | AND **English language**  **AND Human studies** | **23** |
